# Dynamic RNA binding and unfolding by nonsense-mediated mRNA decay factor UPF2

**DOI:** 10.1261/rna.080300.124

**Published:** 2025-07

**Authors:** Jenn-Yeu A. Szeto, Mirella Vivoli Vega, Justine Mailliot, George Orriss, Lingling Sun, Joshua C. Bufton, Kyle T. Powers, Sathish K.N. Yadav, Imre Berger, Christiane Schaffitzel

**Affiliations:** 1 School of Biochemistry, University of Bristol, Bristol BS8 1TD, United Kingdom; 2 School of Chemistry, University of Bristol, Bristol BS8 1TS, United Kingdom; 3 Max Planck Bristol Centre for Minimal Biology, Bristol BS8 1TS, United Kingdom

**Keywords:** nonsense-mediated mRNA decay, up-frameshift protein 2, dynamic RNA–UPF protein complexes, RNA unfolding

## Abstract

Nonsense-mediated mRNA decay (NMD) is an mRNA surveillance pathway involved in translational control and gene expression regulation. Core NMD factors up-frameshift proteins UPF1, UPF2, and UPF3B are conserved from yeast to humans and essential to target mRNAs with a premature stop codon for decay. UPF2 binding to UPF1 activates UPF1's ATPase and helicase activities, and UPF2 binding to UPF3B is important for its association with the exon junction complex and efficient NMD. However, UPF2's association with RNA remains largely uncharacterized. Here, we analyze nucleic acid binding, identifying the first and third MIF4G domains of UPF2 as main RNA-/DNA-binding modules. We find that UPF2's MIF4G domain-3 has RNA annealing activity, while full-length UPF2 unfolds our reporter hairpin RNA structure. We show that UPF2 preferentially binds and stabilizes single-stranded RNA (ss-RNA) in a sequence-independent manner. Concomitant to ss-RNA binding, UPF2 undergoes a distinct conformational change in its otherwise highly dynamic structure. UPF2's RNA binding and unfolding activity may support UPF1's helicase and messenger ribonucleoprotein remodeling activity and, in combination with UPF3B, stabilize UPF1's association with nonsense mRNA.

## INTRODUCTION

Nonsense-mediated mRNA decay (NMD) recognizes and degrades mRNAs with a premature termination codon (PTC) arising from genetic mutations, gene expression errors, or alternative splicing events (Karousis and Mühlemann 2019; Kurosaki et al. 2019; Lejeune 2022). Thereby, NMD protects cells from the potentially harmful effects of C-terminally truncated protein products (Kurosaki et al. 2019). Translation termination at a PTC is recognized by the NMD machinery, which includes up-frameshift proteins UPF1, UPF2, and UPF3B (Karousis and Mühlemann 2019; Kurosaki et al. 2019). Assembly of the NMD machinery allows phosphorylation of UPF1 by the suppressors with morphological effects on genitalia protein 1 (SMG1) kinase complex (Karousis and Mühlemann 2019; Lejeune 2022). Phospho-UPF1 serves as a binding platform for the SMG5–SMG7 heterodimer and SMG6 endonuclease, which cleaves the mRNA near the PTC, initiating decay (Kurosaki et al. 2019). SMG5–SMG7 recruits mRNA decapping and deadenylation enzymes, as well as exonucleases, to degrade the mRNA (Karousis and Mühlemann 2019; Lejeune 2022). In addition to its role in mRNA quality control, NMD also controls 5%–10% of human endogenous transcripts, thus regulating gene expression and shaping essential biological processes in development, differentiation, and stress (Jaffrey and Wilkinson 2018; Nasif et al. 2018; Kurosaki et al. 2019). Loss or inactivation of NMD factors UPF1, UPF2, UPF3B, SMG1, and SMG6 cause lethality during early embryo development (Chousal et al. 2022). Importantly, NMD is implicated in ∼20% of human genetic diseases caused by a single base-pair mutation (Mort et al. 2008), and mutations in NMD factors are associated with neurodevelopmental disorders and various cancers (Jaffrey and Wilkinson 2018; Tan et al. 2022).

UPF1 is the key NMD factor involved in NMD substrate recognition, recycling of terminating ribosomes, remodeling of messenger ribonucleoprotein complexes (mRNP), and initiation of decay (Fiorini et al. 2015; Lee et al. 2015; Serdar et al. 2020; Chapman et al. 2022). UPF1 binds RNA in a non-sequence-specific manner with high affinity when adopting a closed conformation (Cheng et al. 2007; Hogg and Goff 2010; Lee et al. 2015; Karousis and Mühlemann 2019). Binding of UPF2 to UPF1 stabilizes an open conformation of UPF1 with lower mRNA-binding affinity, while enhancing UPF1's RNA helicase and ATPase activities (Clerici et al. 2009; Chakrabarti et al. 2011). Based on magnetic tweezer experiments, UPF1 helicase was suggested to translocate along the mRNA in 5′ to 3′ direction, unwinding mRNA structures and dissociating or displacing RNA-bound proteins (Fiorini et al. 2015). Mutational studies challenged the importance of UPF1's helicase activity for NMD. They showed that NMD is fully supported by UPF1 mutants with poor unwinding/helicase and wild-type ATPase activities, while NMD is impaired by ATPase-deficient UPF1 mutations (Chapman et al. 2022).

NMD factors UPF2 and UPF3B are associated with the exon junction complex (EJC), which is deposited during mRNA splicing 20–24 nt upstream of the exon–exon junction (Melero et al. 2012; Le Hir et al. 2016; Schlautmann and Gehring 2020). The presence of one or more EJCs in the 3′ untranslated region of an mRNA is a strong marker for NMD (Lindeboom et al. 2016). UPF2 and UPF3B are often presented as “adaptor proteins,” bridging UPF1, the SMG1–8–9 kinase complex and the EJC to form a decay-inducing (DECID) complex (Kashima et al. 2006). While both factors are essential for efficient NMD, UPF2-independent and UPF3B-independent NMD pathways have been reported (Gehring et al. 2005, 2009; Chan et al. 2009; Mabin et al. 2018).

**FIGURE 1.**
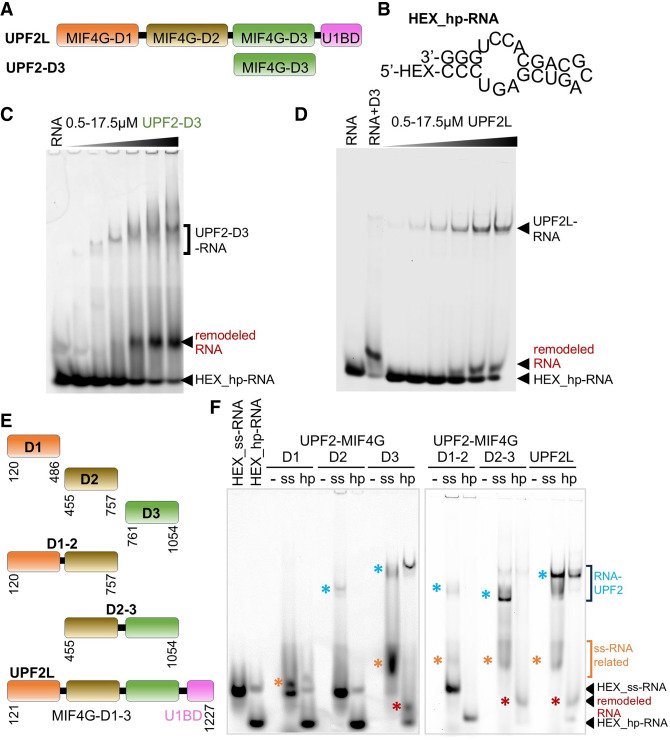
RNA binding of UPF2 variants tested by electromobility shift assays. (*A*) Schematic showing UPF2L (residues 121–1227) and UPF2 MIF4G domain 3 (D3, 761–1054) constructs. (*B*) Hexachlorofluorescein (HEX)-labeled hairpin RNA (hp-RNA) construct used in this study (prediction based on RNAfold web server) (Lorenz et al. 2011). (*C*,*D*) Electromobility shift assays (EMSAs) using HEX_hp-RNA (250 nM) and increasing concentrations of MIF4G-D3 and UPF2L. Remodeled RNA bands appearing in the presence of UPF2 variants are highlighted (red). (*E*) UPF2 constructs used for EMSAs shown in *F*. (*F*) EMSAs using no RNA (−), HEX-labeled ss-RNA (ss), and HEX_hp-RNA (hp) (250 nM each) and UPF2 constructs. Corresponding Coomassie-stained gel sections are shown in Supplemental Figure S1C. Remodeled RNA-containing bands are highlighted with asterisks.

UPF2 is a 148 kDa perinuclear protein that comprises three middle portions of eIF4G (MIF4G) domains and a C-terminal UPF1-binding domain (U1BD) (Fig. 1A). The U1BD adopts a mixed α-helical/β-hairpin when interacting with UPF1's cysteine–histidine-rich (CH) domain (Clerici et al. 2009). UPF2 U1BD binding induces an open conformation of UPF1 with enhanced ATPase and RNA helicase activities (Clerici et al. 2009; Chakrabarti et al. 2011). The third MIF4G domain (MIF4G-D3) of UPF2 is essential for NMD machinery assembly via a tight interaction with UPF3B, which supports UPF2 association with the EJC (Kadlec et al. 2004; Buchwald et al. 2010; Bufton et al. 2022). MIF4G-D3 further binds to SMG1, facilitating the activation of SMG1 kinase activity (Kashima et al. 2006; Clerici et al. 2014; Deniaud et al. 2015). The UPF2 MIF4G-D3 domain also binds RNA (Kadlec et al. 2004; Bufton et al. 2022), whereas, to the best of our knowledge, DNA binding by UPF2 has not been reported. In fact, UPF2 binding to UPF3B induces a conformational change in UPF3B, interfering with RNA-induced oligomerization of UPF3B and delays translation termination by UPF3B (Neu-Yilik et al. 2017; Bufton et al. 2022). More recently, low-affinity RNA binding was shown for a construct comprising UPF2's MIF4G domain-1 and domain-2 (MIF4G-D1–2), while no RNA binding was detected for the U1BD alone, indicating that the three MIF4G domains are responsible for RNA binding (Xue et al. 2023). Notably, this study also showed that RNA binding by UPF1 is weakened through interactions with UPF2, thereby preventing the formation of a stable ternary complex (Xue et al. 2023).

Here, we systematically probed UPF2's ability to bind nucleic acids. UPF2 and the MIF4G-D3 both bind RNA and DNA in their single-stranded (ss) and double-stranded (ds) forms. However, UPF2 has a clear preference for ss-RNA. Based on fluorescence anisotropy measurements, RNA binding by UPF2 is mostly mediated by MIF4G domains 1 and 3 (D1 and D3), with no clear preference for a particular sequence, pyrimidines or purines. Intriguingly, when analyzing UPF2's binding to a model hp-RNA using electromobility shift assays (EMSAs), we discovered that interaction between UPF2 and MIF4G-D3 with hp-RNA changes the RNA conformation. This activity is maintained in the presence of UPF1 and UPF3B. Notably, stable RNA associations are only formed by UPF2–UPF3B and UPF1–UPF2–UPF3B complexes. Using circular dichroism (CD) and fluorescence spectroscopy, we corroborate that UPF2 can unfold the hp-RNA. While UPF2's structure is highly dynamic in the absence of RNA, binding of ss-RNA leads to more compact conformations of UPF2, reminiscent of the conformation reported previously after glutaraldehyde crosslinking (Melero et al. 2012; Lopez-Perrote et al. 2016). Our results suggest that the preferential binding to ss-RNA by UPF2's MIF4G domains, in combination with UPF1–UFP2–UPF3B complex formation, enhances UPF1–UPF2's attachment to nonsense RNA and contributes to RNA unfolding and mRNP remodeling, thereby facilitating mRNA decay.

## RESULTS

### UPF2 binds and remodels structured RNA

We previously reported RNA binding by the paralogs UPF3A and UPF3B using a 24 nt long 5′ HEX-labeled hp-RNA (Fig. 1B; Neu-Yilik et al. 2017; Bufton et al. 2022). This RNA forms a hairpin with bulges, which likely mimics imperfect structures formed by mRNA more closely than a perfect hairpin or a poly(U) oligonucleotide that is single-stranded. A 24 nt RNA was used in this study as longer RNAs show more conformational dynamics that enabled binding of two UPF2 molecules per RNA. In the EMSAs, RNA-induced oligomerization of UPF3B was prevented by MIF4G domain-3 of UPF2 (Bufton et al. 2022). Instead, a stable complex comprising RNA, UPF3B and UPF2's MIF4G-D3 was formed with defined stoichiometry (Bufton et al. 2022). Here, we tested the binding of UPF2 MIF4G-D3 alone with hp-RNA by EMSAs. As expected, based on previous studies (Bufton et al. 2022; Xue et al. 2023), we observed the formation of hp-RNA/MIF4G-D3 complexes (Fig. 1B,C). Surprisingly, addition of increasing concentrations of MIF4G-D3 led to the appearance of a defined, new RNA species giving rise to a band migrating slower in the gel than the hp-RNA input construct (Fig. 1C). Concomitantly, the hp-RNA band disappeared. This indicates that a different, distinct RNA structure is formed in the presence of high concentrations of UPF2's MIF4G-D3.

Therefore, we next tested UPF2L (residues 121–1227), which comprises the three MIF4G domains and U1BD and has the same activity as full-length UPF2 (Chakrabarti et al. 2011). We asked whether UPF2L would also induce the formation of a remodeled RNA species in EMSA gels. A band corresponding to a defined UPF2L/hp-RNA complex was observed with increasing concentrations of UPF2L (Fig. 1D). In addition, a “RNA-only” band appeared with increasing concentrations of UPF2L, with the hp-RNA band becoming less pronounced. This new, remodeled RNA species migrated slower in the gel compared to hp-RNA alone (Fig. 1D). In fact, the position of the remodeled RNA band was not identical to the band observed in the presence of MIF4G-D3, which ran higher in comparison (Fig. 1D). This suggests that a novel RNA structure is formed and released by UPF2L, which is different from the hp-RNA and the structure promoted in the presence of MIF4G-D3 alone. It also implies that the other domains of UPF2 contribute to RNA binding and rearranging the RNA structure.

### MIF4G domains mediate RNA binding of UPF2

We set out to determine which domains of UPF2 were responsible for RNA binding and forming these remodeled RNA species. In addition to the 24 nt hp-RNA, we tested a 24 nt ss-RNA construct derived from the hp-RNA by removing self-complementarity (Supplemental Fig. S1A). The design of the ss-RNA sequence was verified using RNA structure prediction software RNAfold (Lorenz et al. 2011), 3dRNA/DNA (Zhang et al. 2022), and trRosettaRNA (Supplemental Fig. S1A,B; Wang et al. 2023). To identify the UPF2 domains responsible for RNA binding, we expressed six different UPF2 variants (Fig. 1E), comprising the individual MIF4G domains (D1, D2, and D3), combinations of domains 1 and 2 (D1–2) and domains 2 and 3 (D2–3), as well as UPF2L. We did not test UPF2's U1BD, which does not bind RNA as shown previously (Xue et al. 2023).

In EMSA experiments, the ss-RNA runs higher in the gel compared to hp-RNA (Fig. 1F). All UPF2 constructs tested, except for MIF4G-D1, bound ss-RNA as evidenced by protein–RNA complex bands for D2, D3, D1–2, D2–3, and UPF2L constructs (Fig. 1F, blue stars). Additionally, we observed RNA bands (discrete or diffuse) running higher in the gel for constructs D1, D3, D1–2, D2–3, and UPF2L (Fig. 1F, orange stars). This is consistent with complexes between ss-RNA and UPF2 variants dissociating during EMSAs. A defined complex band was observed for hp-RNA/UPF2L and hp-RNA/MIF4G-D3, but not for the other UPF2 constructs (Fig. 1F). For the hp-RNA samples, we observed the formation of a remodeled RNA species in the presence of MIF4G-D3, D2–3, and UPF2L, but not for the D1, D2, or D1–2 constructs (Fig. 1F, red stars). Therefore, it appears that RNA remodeling is specifically dependent on MIF4G domain-3 and only constructs comprising this domain (D3) could generate this novel RNA species.

Finally, ss-RNA or hp-RNA binding does not lead to a detectable upward shift of the protein bands in the EMSA gels for all UPF2 variants (Supplemental Fig. S1C). This indicates that no RNA-induced oligomerization or stable RNA/UPF2 complexes occur under the conditions tested. Taken together, based on our EMSA experiments, it appears that all MIF4G domains of UPF2 contribute to RNA binding to varying degrees, with D3 being essential for RNA structural rearrangements.

### RNA complex formation and remodeling in the presence of UPF3B and UPF1

UPF1 and UPF3B are central protein interaction partners of UPF2. Therefore, we asked whether the RNA binding and remodeling activity of UPF2 is maintained in the presence of these proteins. We previously established that a stable ternary complex comprising RNA, UPF3B, and MIF4G-D3 can be formed (Bufton et al. 2022). To test for the presence of the remodeled RNA species, we incubated UPF3B (residues 41–262) (Bufton et al. 2022) with hp-RNA and added increasing amounts of MIF4G-D3 (Supplemental Fig. S2). In the absence of MIF4G-D3, a defined shift to the RNA–protein complex was observed upon addition of UPF3B, consistent with RNA binding (Hauer et al. 2016; Neu-Yilik et al. 2017; Bufton et al. 2022). However, adding a large excess of UPF3B compared to hp-RNA leads to RNA-induced oligomerization (Bufton et al. 2022). The RNA–UPF3B complex is too large to migrate into the gel and is found in the loading well (Supplemental Fig. S2). UPF3B's oligomerization is prevented by adding increasing amounts of UPF2 MIF4G-D3, leading to the formation of defined MIF4G-D3/UPF3B/hp-RNA and MIF4G-D3/hp-RNA complexes (Supplemental Fig. S2). In addition to these defined complexes, we observe a small hp-RNA band. The “remodeled RNA” species is detected in the presence of UPF3B and UPF2 MIF4G-D3, as well as UPF2 MIF4G-D3 alone, but is not present in UPF3B alone (Supplemental Fig. S2). Thus, UPF2 MIF4G-D3 appears to be active in the presence of UPF3B in remodeling the hp-RNA structure.

**FIGURE 2.**
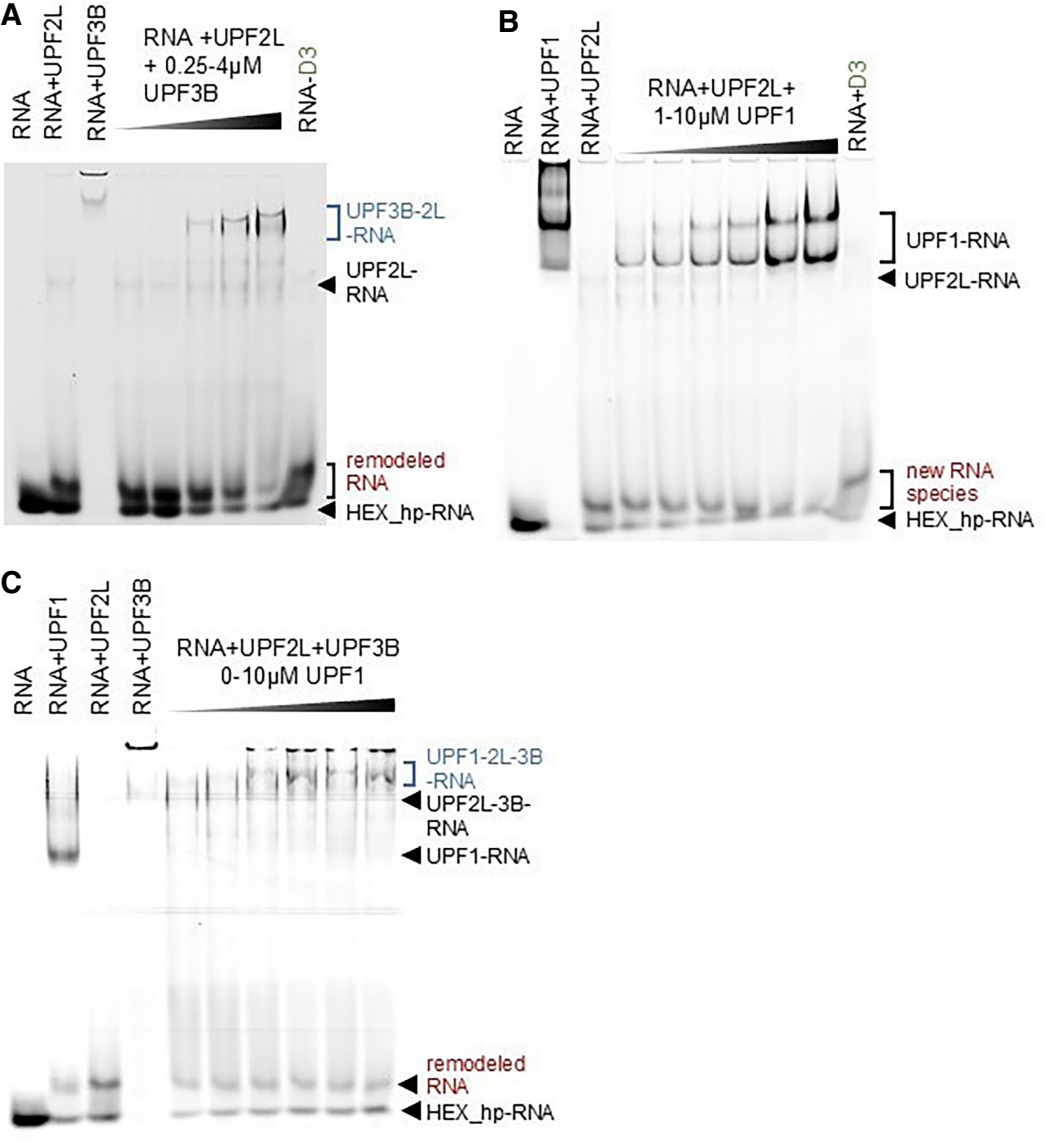
UPF2L complexes and RNA remodeling in the presence of UPF3B or UPF1. (*A*) EMSAs using 250 nM HEX_hp-RNA in all lanes. As control, HEX_hp-RNA was incubated with 10 µM UPF2L alone or 4 µM UPF3B alone. A premix of 10 µM UPF2L with HEX_hp-RNA was supplemented with increasing concentrations of UPF3B. (*B*) EMSAs using HEX_hp-RNA (250 nM) incubated with 10 µM UPF1 alone or 10 µM UPF2L alone. A premix of HEX_hp-RNA with 10 µM UPF2L was supplemented with increasing concentrations of UPF1. (*C*) EMSAs using 250 nM HEX_hp-RNA incubated with 10 µM UPF1 alone, 10 µM UPF2L alone, or 1.6 µM UPF3B alone. A premix of HEX_hp-RNA with 10 µM UPF2L and 4 µM UPF3B was supplemented with increasing concentrations of UPF1. (*A*–*C*) Remodeled RNA bands appearing in the presence of UPF2L or MIF4G domain-3 (control) are highlighted in red; RNA–protein complexes comprising UPF2L are highlighted in blue.

Next, we tested UPF2L's RNA binding/remodeling in the presence of UPF3B. Again, a remodeled RNA species is formed with UPF2L and a UPF2L/UPF3B heterodimer (Fig. 2A). We detect the appearance of the “remodeled RNA species” with UPF2L alone and in the presence of UPF2L/UPF3B complex. As more RNA is bound to UPF2L/UPF3B proteins, the hp-RNA band and the remodeled RNA species become weaker but do not disappear entirely, despite excess of protein. We conclude that UPF2L remains active in RNA remodeling in the presence of UPF3B. Concomitantly, the affinity to RNA appears to be highest for UPF3B, and higher for the UPF2L/UPF3B heterodimer compared to UPF2L alone, leading to a significant but not complete up-shift of the RNA in the EMSA gels.

We then tested whether the binding of UPF1 to UPF2L would interfere with UPF2's RNA remodeling activity. UPF1 (residues 115–914, comprising the CH and helicase domains) bound the RNA efficiently as evidenced by an up-shift of the hp-RNA band (Fig. 2B). UPF2L incubation with hp-RNA led to the appearance of the “remodeled RNA” species as observed previously (Fig. 1D). Addition of increasing amounts of UPF1 to UPF2L/RNA resulted in the appearance of an UPF1/RNA complex. The “remodeled RNA” did not disappear, but it runs closer to the hp-RNA band when UPF1 is present in high concentrations. We note that there is a faint band of “remodeled RNA” species visible in the UPF1 control as well, indicating that this somewhat smaller band observed at high UPF2L and high UPF1 concentrations could have been released from UPF1 or from UPF2L (Fig. 2B). A UPF2L/UPF1/RNA complex was not observed. Notably, UPF1 appears to have decreased affinity for RNA in the presence of UPF2L (Fig. 2B). These observations agree with previous reports showing that UPF2 destabilizes RNA binding by UPF1, confirming that ternary UPF2L/UPF1/RNA complexes are unstable and transient in nature (Chamieh et al. 2008; Xue et al. 2023).

Finally, we tested RNA in the presence of all three UPF proteins. UPF2L and UPF1 incubation with hp-RNA led to the appearance of the “remodeled RNA species” (Fig. 2C). Also, UPF3B–UPF2L–RNA complexes were formed, resolving the large RNA–UPF3B oligomers. Addition of increasing concentrations of UPF1 to UPF2L/UPF3B–RNA complexes resulted in an increased up-shifting of the RNA into a UPF1/UPF2L/UPF3B–RNA complex. At the same time, the hp-RNA band and the “remodeled RNA species” band remained constant (Fig. 2C).

Taken together, the addition of UPF3B, or UPF3B and UPF1, to UPF2L leads to increased RNA–protein complex formation, but free hp-RNA and the remodeled RNA species can still be detected. This suggests that UPF2L can remodel hp-RNA in the presence of UPF1 and UPF3B, and that UPF3B is required for stable complexes containing RNA and UPF2.

### UPF2L preferentially binds ss-RNA

**FIGURE 3.**
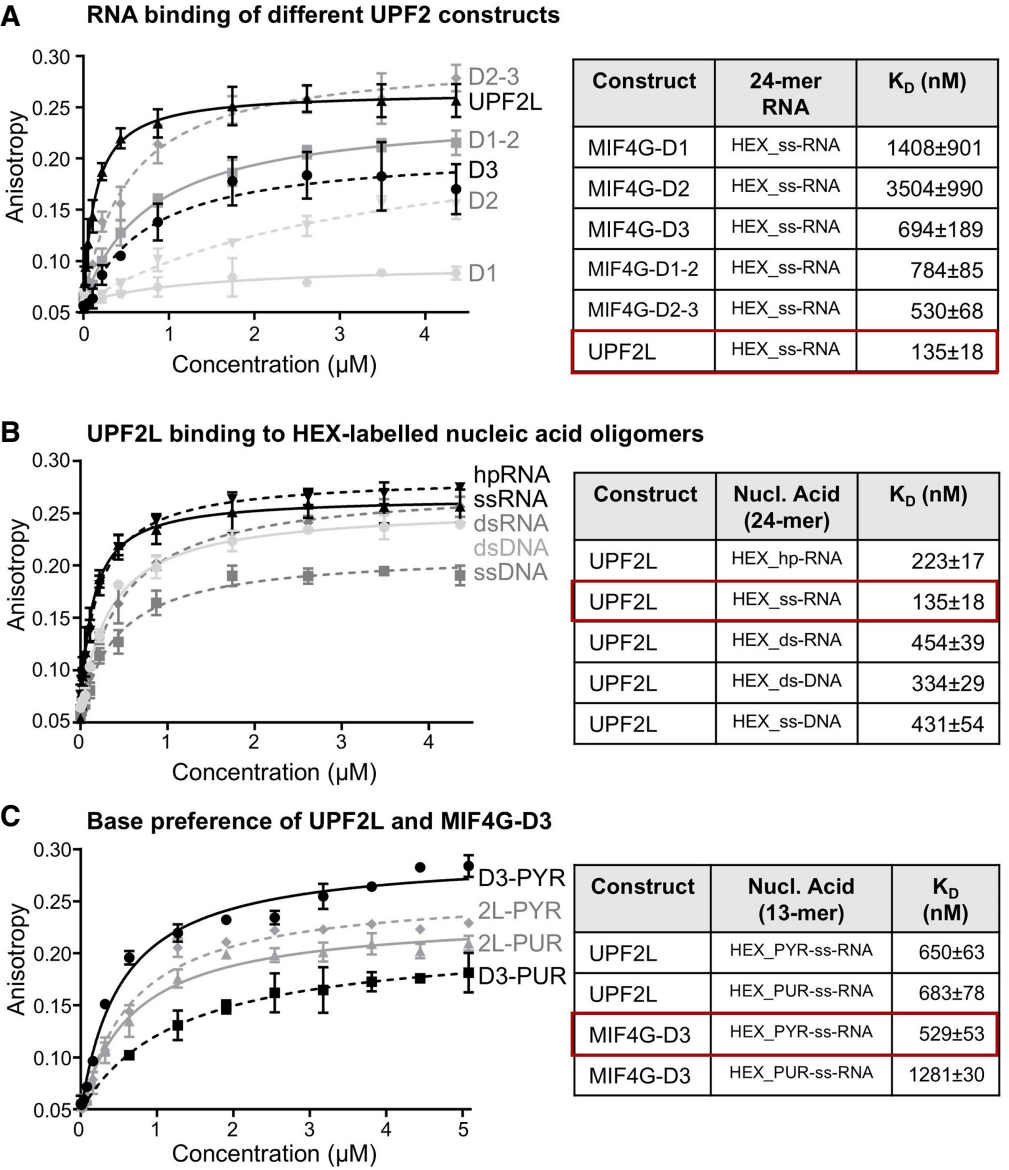
Nucleic acid binding of UPF2 variants determined by fluorescence anisotropy. (*A*, *left*) Fluorescence anisotropy binding curves of UPF2 constructs to HEX-labeled 24 nt ss-RNA. (*Right*) Measurements were performed in triplicate and error bars plotted via standard deviation before fitting a single component binding equation to calculate *K*_D_ values. (*B*) Binding curves of UPF2L to HEX-labeled 24 nt ss- and ds-RNA and DNA constructs are shown at the *left*. (*C*) Binding curves of UPF2L and MIF4G-D3 to HEX-labeled 13 nt pyrimidine-rich (PYR) or purine-rich (PUR) ss-RNA constructs are shown at the *left*. (*A*–*C*) *K*_D_ values are listed in the table at the *right*) with highest affinity highlighted by a red box. Sequences are listed in Supplemental Table S1.

To quantify the contribution of the individual domains of UPF2 and understand the nature of UPF2's interaction with nucleic acids, we analyzed DNA and RNA binding by fluorescence anisotropy. We first determined the affinity of the MIF4G domains alone and in combinations (Fig. 1E) for ss-RNA (Supplemental Fig. S1B). The dissociation constants (*K*_D_) for UPF2 MIF4G-D1, D2, and D3 constructs were determined as 1.4 µM, 3.5 µM, and 694 nM, respectively (Fig. 3A). This indicates that D1 and D3 are mainly responsible for RNA binding with D3 being the key domain. UPF2 MIF4G-D1–2 and D2–3 bound ss-RNA with a *K*_D_ of 784 and 530 nM, respectively. This is consistent with a minor contribution of D2 toward binding of ss-RNA by MIF4G-D3. In D1–D2, a more significant increase in affinity for binding ss-RNA is detected, indicating some cooperativity between D1 and D2 in RNA binding (Fig. 3A). Combining the binding sites of all three MIF4G domains, UPF2L had an affinity of 135 nM for ss-RNA. These *K*_D_ values are in good agreement with previously determined *K*_D_ values of 609 nM for a MIF4G-D3-U1BD variant and 761 nM for a D1–2 variant using a 12-nt poly(U) RNA (Xue et al. 2023). The agreement between the previously determined *K*_D_ values and our results also indicates that UPF2's ss-RNA binding is sequence-independent. Taken together, these experiments show that in UPF2, all three MIF4G domains can interact with RNA, resulting in a *K*_D_ value of 135 nM, which is compatible with mRNA binding by UPF2 in cells.

Subsequently, we investigated whether UPF2 preferentially binds RNA or DNA in their ds or ss forms. The sequences of the nucleic acids used for fluorescence anisotropy are listed in Supplemental Table S1. First, we tested the 24 nt hp-RNA, the 24 nt ss-RNA, and a 24 nt ds-RNA. Fluorescence anisotropy experiments reveal a clear difference in the binding affinities between ds-RNA and ss-RNA binding, with *K*_D_ values of 454 and 135 nM, respectively (Fig. 3B). As expected, the hp-RNA, which comprises both ds regions and ss bulges (Supplemental Fig. S1A,B), bound with intermediate affinity to UPF2L (*K*_D_ of 223 nM), (Fig. 3B). The difference in affinity between UPF2L binding to ds-DNA and ss-DNA (sequences in Supplemental Table S1) was less obvious, resulting in *K*_D_ measurements of 334 and 431 nM, respectively. This indicates a slight preference of UPF2 for binding ds-DNA (Fig. 3B). Clearly, UPF2L preferentially binds to ss-RNA as evidenced by its highest affinity (135 nM) for this construct in our experiments.

Next, we asked whether we could identify sequence preferences for UPF2L–RNA binding. We tested two 13 nt RNA sequences that were either purine-rich or pyrimidine-rich (Supplemental Table S1). We could not identify a significant difference in RNA binding of UPF2L by fluorescence anisotropy as the pyrimidine-rich and purine-rich sequences bound RNA with very similar *K*_D_ values of 650 and 683 nM, respectively (Fig. 3C). The lower affinity of UPF2L toward these RNA oligonucleotides was due to the shorter RNA oligonucleotide tested; 13 nt versus 24 nt. In contrast, MIF4G-D3 displayed a strong preference for pyrimidine-rich RNA with a *K*_D_ of 529 nM versus a *K*_D_ of 1.28 µM for purine-rich RNA (Fig. 3C). Here, the difference in *K*_D_ values for MIF4G-D3 ss-RNA binding between the two experiments (694 versus 529 nM for the 13 nt versus 24 nt ss-RNA) appears to originate from the different sequences and not the length of the oligonucleotide. A significant decrease in affinity for shorter RNAs (<13 nt) was observed for MIF4G-D3, with very weak binding to a 9-mer RNA oligonucleotide (Supplemental Fig. S3). This suggests that high-affinity RNA binding by UPF2 MIF4G-D3 requires an RNA sequence length of at least 13 nt with a preference for pyrimidine-rich sequences.

In summary, UPF2 can bind DNA and RNA in their ss and ds form. UPF2 preferentially binds ss-RNA oligonucleotides longer than 13 nt, and we could not identify any sequence preference or preference for purines or pyrimidines for UPF2's nucleic acid binding.

### UPF2 remodels structured RNA

**FIGURE 4.**
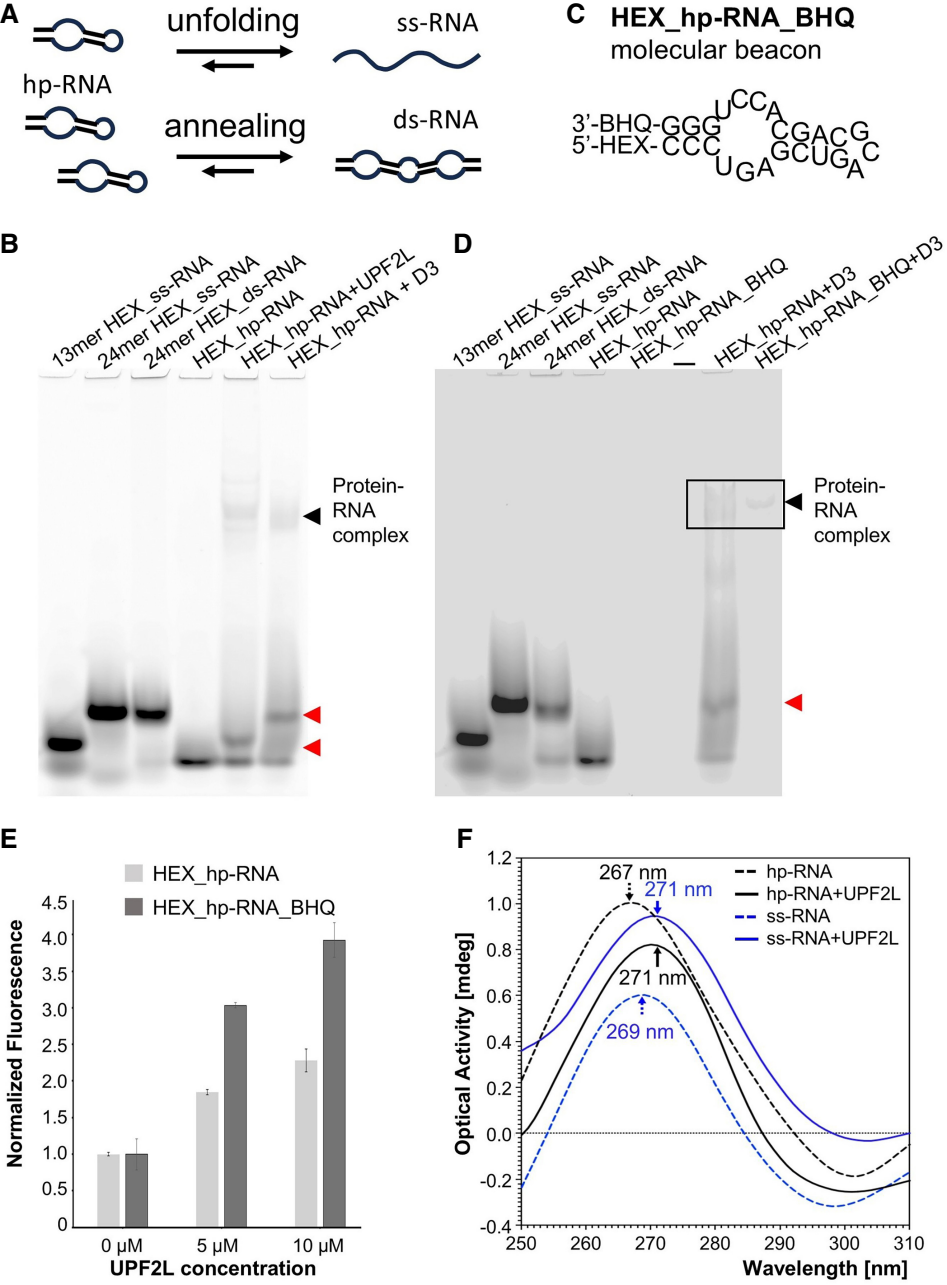
RNA structural changes mediated by UPF2L. (*A*) A schematic illustrating potential UPF2-mediated RNA annealing or RNA unfolding activities. (*B*) EMSAs using HEX-labeled ss-RNA oligonucleotides of 13 and and 24 nt, HEX_ds-RNA, and HEX_hp-RNA as controls. Incubation of HEX_hp-RNA with UPF2L or MIF4G-D3 produces remodeled RNA bands running at similar height as HEX-labeled 13 nt ss-RNA and 24 nt ss-RNA/ds-RNA, respectively (red arrows). (*C*) Molecular beacon hp-RNA (HEX_hp-RNA_BHQ) construct labeled 5′ with HEX dye and 3′ with a blackhole quencher 1 (BHQ-1). (*D*) EMSAs using same controls as in *B*, 250 nM molecular beacon, and MIF4G-D3 incubated with HEX_hp-RNA or molecular beacon. The remodeled RNA band is highlighted (red arrow). (*E*) Normalized fluorescence signal of HEX_hp-RNA (light gray bars) and molecular beacon (dark-gray bars) in the presence of UPF2L compared to RNA only (no UPF2L). Experiments were performed in duplicate. (*F*) Changes in hp-RNA and ss-RNA tertiary structure upon addition of 10 μM UPF2L monitored by CD spectroscopy. (Black dashed line) hp-RNA, (blue dashed line) ss-RNA, (black line) hp-RNA spectrum subtracted by UPF2L spectrum, (blue line) ss-RNA spectrum subtracted by UPF2L spectrum. Peak wavelengths are indicated. For reference: ss poly-uridine peaks at 272 nm.

In our EMSA experiments, we observed the appearance of a new, remodeled band of RNA in the presence of UPF2L and MIF4G-D3 (Fig. 1C,D). However, it remained unclear whether this remodeled RNA band corresponds to ss, ds, or alternatively/partially folded RNA. Unfolding of the RNA hairpin would result in ss-RNA, which is expected to migrate higher in a gel due to its less compact conformation. Given the high complementarity between hp-RNA sequences, UPF2L could also anneal two hp-RNA molecules to form ds-RNA (Fig. 4A). In the EMSA, ds-RNA is also expected to run higher in the gel compared to intramolecularly folded hp-RNA. Annealing of complementary RNAs can occur spontaneously or be accelerated by a protein (Fig. 4A). Such a “protein annealer” can bind one or both RNAs, alter the RNA structure, and enhance the local concentration of RNA and thus the probability of ds-RNA formation (Rajkowitsch et al. 2007). In fact, the three MIF4G domains of UPF2 offer at least two RNA-binding sites in MIF4G-D3 and D1–D2 (Fig. 3A). By binding two RNA molecules, likely in their ss form, UPF2 could thus facilitate annealing of two RNA molecules into a single ds-RNA due to their proximity.

To investigate this, we first performed EMSAs to test for unfolding or annealing. Using varying RNA lengths as size markers in EMSA gels, we loaded HEX-labeled 13 nt ss-RNA, 24 nt ss-RNA, 24 nt ds-RNA, and 24 nt hp-RNA (Fig. 4B). Incubation of the hp-RNA with UPF2L leads to a band that ran similar to the 13 nt ss-RNA in EMSAs, whereas incubation with MIF4G-D3 resulted in a band running at approximately the same height of ss-RNA and ds-RNA (Fig. 4C). This suggests that MIF4G-D3 fully unfolds or anneals the hp-RNA. In contrast, UPF2L binding to hp-RNA results in a partially unfolded RNA species.

To discriminate between RNA annealing and RNA unfolding by UPF2L and MIF4G-D3, we tested a molecular beacon hp-RNA construct with a HEX dye at the 5′-end and a BHQ-1 at the 3′-end (Fig. 4C; Tyagi and Kramer 1996). This molecular beacon was used in EMSAs and fluorescence spectroscopy to determine changes in fluorescence upon addition of UPF2L. Unfolding of RNA is expected to increase the fluorescence of the molecular beacon as the HEX dye would no longer be in close proximity to the BHQ-1 quencher. In contrast, RNA annealing of the hp-RNA should not alter the fluorescence signal, since the 3′ BHQ-1 of the annealed complementary RNA strand will quench the 5′ HEX dye.

We performed EMSAs using the same size markers as before (Fig. 4B,D). As expected, we could not detect a signal for the molecular beacon, confirming quenching of the HEX dye. In the presence of MIF4G-D3, a weak band was observed for the molecular beacon at the height of the RNA–protein complex (Fig. 4D). This indicates that the molecular beacon hp-RNA is unquenched when bound to MIF4G-D3. No band was observed at the height of the remodeled RNA species (Fig. 4D). This suggests that the molecular beacon is still quenched and thus in a ds rather than ss conformation. We conclude that MIF4G-D3 exhibits RNA annealing activity. The same experiment using UPF2L did not show any fluorescent signal for the UPF2L–RNA complex or RNA alone in the EMSA gel and thus was not conclusive (not shown). Therefore, we decided to determine any conformational change by using fluorescence spectroscopy which is an equilibrium measurement method and potentially more sensitive in detecting unquenched RNA.

Fluorescence spectroscopy would reveal any changes in the environment of the HEX dye in the molecular beacon and in the hp-RNA constructs in the presence of UPF2L, leading to observed structural changes of the RNA in EMSA gels. In the absence of UPF2L, background fluorescence was detected, indicating that not all HEX_hp-RNA and molecular beacon probes are in a hairpin conformation (Supplemental Fig. S4). In agreement, we occasionally observed a second band for the “HEX_hp-RNA only” control, running slower in the EMSA gel (Fig. 1C,F). Addition of increasing concentrations of UPF2L led to increased fluorescence signals suggesting spatial separation of the fluorescent dye and the BHQ-1 quencher in the molecular beacon, consistent with unfolding (Fig. 4E; Supplemental Fig. S4). Notably, we also observed an increase in fluorescence of the HEX_hp-RNA control upon addition of UPF2L (Fig. 4E). This is likely due to protein-induced fluorescence enhancement (PIFE), a photo-physical effect where binding of a protein in proximity leads to increased fluorophore intensity (Hwang and Myong 2014). However, the relative increase in fluorescence in the presence of UPF2L is higher for the HEX_hp-RNA_BHQ/molecular beacon compared to the HEX_hp-RNA signal increase (Fig. 4E). We conclude that the observed fluorescence increase for the molecular beacon in the presence of UPF2L is due to PIFE *and* due to unquenching of the HEX dye caused by a conformational change of the molecular beacon RNA structure toward ss-RNA.

To further confirm the formation of ss-RNA rather than ds-RNA in the presence of UPF2L, CD spectroscopy was performed, allowing detection of changes in tertiary RNA structure. In its A-form, ds-RNA is known to produce in CD spectra a positive peak at ∼260 nm and a negative peak near 210 nm, whereas ss-RNA [such as poly(U)] displays a positive peak at ∼272 nm (Chauca-Diaz et al. 2015; Szpotkowski et al. 2023). Our 24 nt ss-RNA displayed a positive peak at 269 nm, indicating it is not completely unstructured, conflicting with computational predictions (Fig. 4F; Supplemental Fig. S1A,B). The 24 nt hp-RNA containing ds-RNA and ss bulges showed a peak at 267 nm in CD spectra (Fig. 4F). This peak value agrees with a small fraction of unfolded RNA. In the presence of excess UPF2L, we observed a shift of the ss-RNA peak from 269 to 271 nm after subtracting the CD curve of UPF2L protein only (Fig. 4F), in agreement with the peak value described for poly(U) ss-RNA. This indicates that UPF2L binding stabilizes the ss-RNA conformation. Similarly, a shift is observed for the hp-RNA from 267 to 271 nm in the presence of excess UPF2L (Fig. 4F) indicating formation of ss-RNA. In summary, the results from CD spectroscopy and molecular beacon fluorescence spectroscopy confirm that UPF2L opens hp-RNA structures and stabilizes ss-RNA.

### UPF2L structural changes upon RNA binding

We subtracted the CD curves of hp-RNA and ss-RNA from the CD curves of UPF2L/hp-RNA and UPF2L/ss-RNA, respectively, to identify any changes in UPF2L structure upon RNA binding. Notably, the CD spectra for UPF2L alone, UPF2L + hp-RNA and UPF2L + ss-RNA showed clear differences between the three samples in the region of 210–230 nm (Supplemental Fig. S5). While all three spectra displayed a negative peak at a wavelength of ∼222 nm characteristic of α-helical secondary structure, the ellipticity intensity at the 222 nm peak was different for the three samples. A ∼3-times stronger negative signal was observed for the UPF2L + ss-RNA complex compared to UPF2L alone (Supplemental Fig. S5). The UPF2L + hp-RNA sample signal amplitude at 222 nm was approximately half of UPF2L alone (Supplemental Fig. S5). The high concentration of UPF2L required for the experiment (10 µM) precluded further characterization of the secondary structure change. Nonetheless, these results indicate that UPF2L changes its structure when binding RNA and becomes more α-helical when in a complex with ss-RNA.

### RNA binding induces more compact UPF2L conformations

**FIGURE 5.**
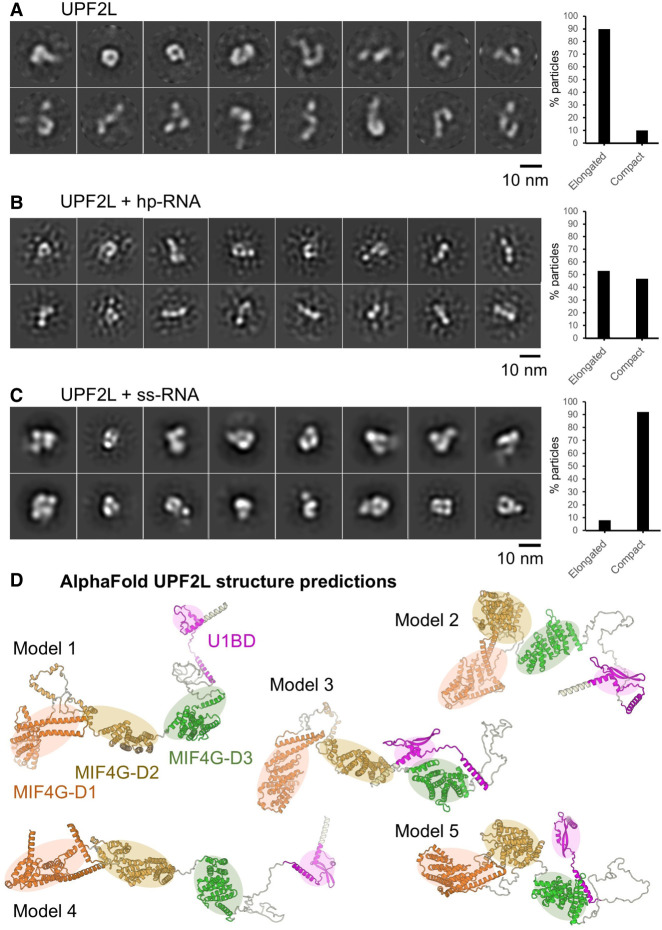
Dynamics and conformational change of UPF2L upon RNA binding. (*A*) Negative-stain EM 2D class averages of UPF2L alone. (*B*) Negative-stain EM 2D class averages of UPF2L incubated with a fourfold molar excess hp-RNA, leading to more V/U shaped particles. (*C*) Negative-stain EM 2D class averages of UPF2L with a fourfold molar excess of ss-RNA, leading to more defined, compact particles. (*A*–*C*) Scale bar, 10 nm. (*Right*) Plot showing the percentage of particles classifying into elongated versus compact, U/V-shaped 2D classes. (*D*) Relaxed AlphaFold2 models of UPF2L (without RNA) showing highly dynamic regions between the individual MIF4G domains and the UPF1-binding domain ([orange] D1, [brown] D2, [green] D3, [magenta] U1BD), agreeing with conformational dynamics and mostly elongated particles observed in *A* and *B*.

To further investigate the structural changes in UPF2L upon RNA binding observed by CD spectroscopy, we performed negative-stain electron microscopy (EM) and 2D classification (Fig. 5A–C; Supplemental Fig. S6A–C). In the 2D class averages of UPF2L alone, we observed a huge variability in shapes, ranging from elongated L and S shapes to more compact V and U shapes, including a closed (doughnut-like) shape (Fig. 5A). The dimensions of the particles range from 10 nm for the more compact shapes to almost 20 nm for the elongated shapes. For UPF2L alone ∼90% of the particles belong to classes that show elongated L/S-shaped particles (Supplemental Fig. S6D). A similar variation in sizes and shapes was observed for the UPF2L sample that was incubated with a fourfold molar excess of hp-RNA (Fig. 5B). However, ∼47% of particles belong to classes with more compact U/V-shapes indicating a conformational change in UPF2L upon hp-RNA binding (Supplemental Fig. S6D). Incubation of UPF2L with fourfold molar excess of ss-RNA led to more defined particles in the micrographs (Supplemental Fig. S6C) and in the 2D class averages (Fig. 5C). Approximately 92% of particles adopt U- or V-shaped compact conformations in the presence of ss-RNA (Supplemental Fig. S6D). Remaining flexibility of the more compact UPF2L/ss-RNA particles precluded a high-resolution cryo-EM study. The compact U/V-shaped particles observed in the current work resemble previously reported low-resolution UPF2 cryo-EM structures where UPF2 was crosslinked with glutaraldehyde prior to single-particle analysis (Melero et al. 2012; Lopez-Perrote et al. 2016). Taken together, our negative-stain EM results and our observations from CD spectroscopy both show that UPF2L changes its structure upon binding hp-RNA and ss-RNA. More compact conformations are adopted in complex with RNA, reminiscent of glutaraldehyde-crosslinked UPF2.

We note that several UPF2L dimers with compact conformations were also observed in the sample with ss-RNA in negative-stain EM and 2D class averages, despite an excess of ss-RNA (Supplemental Fig. S7A). We wondered whether the 24 nt ss-RNA could accommodate the binding of multiple UPF2L molecules. However, our *K*_D_ measurements indicate that shorter RNAs are bound by UPF2L with lower affinity (13 nt), (Fig. 3C). Therefore, we asked whether two ss-RNA molecules could interact by partial annealing. For the computational analysis, we artificially linked two molecules via a U_10_ or A_10_ linker. RNAfold (Lorenz et al. 2011), MXfold2 (Sato et al. 2021), and 3dRNA/DNA (Zhang et al. 2022) predict the formation of a short RNA-helix and thus possible dimerization, whereas trRosettaRNA (Wang et al. 2023) does not predict any interaction between the ss-RNA molecules (Supplemental Fig. S7B). We conclude that the UPF2L/ss-RNA dimers observed in EM are likely mediated by ss-RNA dimers.

Finally, AlphaFold2 (Mirdita et al. 2022) and Rosetta Relax (Tyka et al. 2011) were used to predict the structure of the UPF2L protein (Fig. 5D). Predicted models highlight a flexible, rather elongated arrangement of the MIF4G domains and U1BD (Fig. 5D), recapitulating the L- and S-shapes of UPF2L observed in the 2D class averages from negative-stain EM (Fig. 5A). We used AlphaFold3 (Abramson et al. 2024) to obtain models of UPF2L/ss-RNA complexes. Unfortunately, the confidence of the best five models is very low for UPF2L/ss-RNA complexes with interchain predicted template modeling (ipTM) score values ranging from 0.21 to 0.27. Such low ipTM values mean that we cannot reliably predict interfaces between UPF2L and RNA, likely due to non-canonical protein–RNA interaction and lack of training data (Hennig 2025).

Taken together, our results indicate a flexible domain arrangement of UPF2L in the absence of RNA, which shifts to more compact conformations when a complex is formed with hp-RNA or ss-RNA.

## DISCUSSION

In this study, we analyzed RNA binding by UPF2 and its domains. Our experiments show that UPF2L can bind DNA but preferentially binds ss-RNA with no apparent sequence specificity. We discovered that UPF2L and MIF4G-D3 alter the structure of hp-RNA. Notably, the novel RNA species observed in EMSAs are not identical for these proteins. The remodeled RNA species produced by MIF4G-D3 migrates slower in the gel compared to the species observed in the presence of UPF2L. This indicates that additional domains of UPF2 also play a role in nucleic acid binding and remodeling. In fact, fluorescence anisotropy experiments demonstrate that all three MIF4G domains can interact with ss-RNA. MIF4G-D1 has an affinity of 1.4 µM, and MIF4G-D2 has a much weaker affinity of 3.5 µM. However, in combination, D1–D2 bind ss-RNA with an affinity of ∼800 nM. This agrees with the *K*_D_ for MIF4G-D1–2 determined in a previous study testing shorter U_15_ RNA (Xue et al. 2023). MIF4G-D3 alone binds the model RNA with ∼700 nM affinity, and in combination with D2 the dissociation constant moderately decreased to ∼530 nM, confirming a small contribution of D2. UPF2L binds ss-RNA with a *K*_D_ of 135 nM, in agreement with the avidity expected for at least three RNA-binding sites. This indicates that UPF2 is an RNA-binding protein under physiological conditions, unless its binding to nucleic acids would be prevented by complex formation with other proteins.

When we tested UPF2's RNA interactions in the presence of UPF3B and UPF1 using EMSAs, we found that a tertiary complex can be formed by UPF2L/UPF3B/hp-RNA and that an additional RNA species running higher than hp-RNA is still observed in the presence of UPF2L and UPF3B. Addition of UPF1 to UPF2L/hp-RNA complexes led to increased UPF1/RNA complex formation. However, the remodeled RNA species is still observed, indicating that UPF2L can remodel the unbound hp-RNA. Notably in EMSAs, the gel shift observed for the UPF1/hp-RNA is significantly stronger than the shift observed for UPF1 in the presence of UPF2L and hp-RNA indicating decreased affinity for RNA. There is also no sign of a UPF1/UPF2L/hp-RNA complex in EMSA gels, confirming that UPF1 binds either UPF2 or RNA and that the ternary complex is unstable (Chamieh et al. 2008; Chakrabarti et al. 2011; Xue et al. 2023). When UPF1 was added to UPF2L/UPF3B/hp-RNA complexes, an additional band corresponding to a UPF1/UPF2L/UPF3B/hp-RNA complex was detected, indicating that UPF3B is important for stable RNA association (Fig. 2).

UPF2L does not form stable RNA–protein complexes in EMSA gels, a non-equilibrium method. Free ss and hp-RNAs are mostly released from UPF2L in EMSA, even in the presence of excess UPF2L. This also applies to all UPF2 variants tested. We suggest that UPF2L and MIF4G-D3 dynamically bind and release RNA. Concomitantly, they remodel the hp-RNA and release a remodeled RNA species in a process that does not consume energy. Notably, the remodeled RNA band observed in the presence of UPF2L runs at the same height as 13 nt ss-RNA and not at the height of 24 nt ss-RNA, suggesting that the RNA is not completely unstructured/unfolded. In marked contrast, in the presence of MIF4G-D3, a band corresponding to 24 nt ss-RNA or 24 nt ds-RNA is detected by EMSAs. This remodeled RNA band is completely quenched when a molecular beacon probe is used, indicating that it corresponds to ds-RNA, while a small fraction of unquenched ss-RNA is detected in a MIF4G-D3/RNA complex. We conclude that MIF4G-D3 exhibits an annealing activity, bringing two unstructured RNA molecules in close proximity to form ds-RNA. Dequenching of the molecular beacon was also observed in the presence of UPF2L in fluorescence spectroscopy (Fig. 4). This indicates that the HEX fluorescence label has a larger distance to the BHQ-1 quencher and, accordingly, the hp-RNA is unstructured in that region. This finding was further corroborated by CD spectroscopy showing an increased proportion of ss-RNA in the presence of UPF2.

CD spectroscopy indicated that UPF2L is highly flexible (Supplemental Fig. S5). In contrast, an increase in secondary structure was observed in the presence of the 24 nt ss-RNA, suggesting UPF2L/ss-RNA complex formation and a conformational change in the protein. Consistently, we observe that UPF2L adopts many different conformations in negative-stain EM 2D class averages (Fig. 5). Similarly, AlphaFold2 (Mirdita et al. 2022) and Rosetta Relax (Tyka et al. 2011) predict a very flexible domain arrangement with virtually no interactions between the individual domains of UPF2. In contrast, more compact U/V-shaped UPF2L conformations are observed in the presence of hp-RNA and ss-RNA in negative-stain EM 2D class averages. We observe a lower percentage (47%) of hp-RNA/UPF2L compact conformations compared to U/V-shaped ss-RNA/UPF2L complexes (90%), consistent with the *K*_D_ values (Fig. 3B) and the observation that hp-RNA/UPF2L complexes tend to be more unstable in EMSAs (Fig. 1F). Thus, our data support (i) conformational changes of UPF2L in the presence of RNA and (ii) more transient hp-RNA/UPF2L complex formation or distinct binding modes of UPF2L to hp-RNA versus ss-RNA. We hypothesize that UPF2L cannot stably bind hp-RNA with all three MIF4G domains. We note that the pseudo-atomic model of MIF4G domains 2 and 3 from X-ray crystallography (Clerici et al. 2014) could not be fitted into the negative-stain EM density of glutaraldehyde-crosslinked UPF2 (Melero et al. 2012), indicating different conformations. This further supports that UPF2 domains do show no interactions with each other and UPF2 is a highly dynamic protein.

**FIGURE 6.**
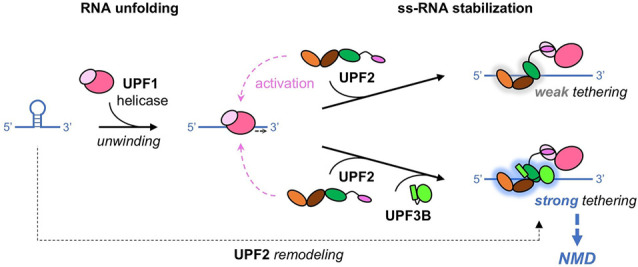
Model of UPF2–RNA interactions. Our data suggest a highly dynamic UPF2 structure and different RNA complex formation in the presence and the absence of UPF1 and/or UPF3B. UPF1 is activated by UPF2 and then weakly tethered to RNA. UPF3B can stabilize UPF1–UPF2's association with RNA, and UPF2 can support UPF1's RNA unfolding activity, corroborating mRNA degradation.

We find that UPF2 preferentially binds and stabilizes unstructured RNA. Moreover, UPF2 can unfold weakly structured RNA, also in the presence of UPF3B. Given that UPF2 is a conserved NMD factor that interacts with and activates UPF1 helicase (Kadlec et al. 2004; Chakrabarti et al. 2011; Fiorini et al. 2015; Serdar et al. 2016, 2020), this UPF2 activity is likely to be relevant in the context of mRNP remodeling and ultimately mRNA decay. It is conceivable that UPF2 binds unstructured mRNA after UPF1 helicase has unwound it (Fig. 6). Thus, UPF2 could prevent re-folding of mRNA by binding it and/or contribute to unfolding mRNA structures, in synergy with UPF1 RNA helicase.

Moreover, UPF2 and UPF3B provide additional RNA-binding sites to the NMD machinery, facilitating the association of UPF1 with a nonsense mRNA substrate. UPF2 also supports UPF1's activation and phosphorylation by SMG1 kinase (Kashima et al. 2006; Clerici et al. 2009, 2014; Chakrabarti et al. 2011; Deniaud et al. 2015). The additional RNA-binding sites of UPF2 are likely not sufficient to ensure that activated UPF1 remains associated with the nonsense RNA because the ternary complex is unstable (Fig. 2B; Chamieh et al. 2008; Chakrabarti et al. 2011). However, the combination of UPF2 and UPF3B leads to more stable ternary complexes with RNA (Fig. 2). UPF2/UPF3B complexes could thus ensure a robust activation of UPF1 and its stable association with a nonsense mRNA (Fig. 6). In fact, the binding and ATPase-dependent dissociation of UPF1 from RNA was found to be important for NMD target discrimination (Lee et al. 2015; Chapman et al. 2022; Fritz et al. 2022). UPF2/UPF1 complex formation and concomitant activation of UPF1 ATPase activity are suggested to contribute to protecting non-target mRNAs because the UPF2-induced destabilization of RNA binding could promote UPF1 dissociation and support efficient recycling of UPF1 from the non-target RNA (Chapman et al. 2024). Conversely, NMD factors should tether activated UPF1 to the target nonsense mRNA for efficient decay, reminiscent of tethering assays of NMD factors to the 3′ UTR of reporter mRNAs, which efficiently initiate NMD (Lykke-Andersen et al. 2000; Gehring et al. 2008). A stable UPF1 association after ATPase activation could be achieved by UPF2/UPF3B complexes (Fig. 6). In fact, the MIF4G domains of UPF2 can bind RNA together with the N-terminal UPF3B domains (Fig. 2; Bufton et al. 2022). We note that individual nucleotide resolution UV crosslinking and immunoprecipitation (iCLIP) experiments indicated that UPF3B can interact with RNA in complex with EJCs and in a non-EJC context (Hauer et al. 2016), the latter may be essential for EJC-independent NMD (Bühler et al. 2006; Metze et al. 2013). In summary, we propose that stable association of activated UPF1 with the nonsense RNA could be achieved by RNA binding of the UPF2/UPF3B complex, alone or when associated with EJCs, to ensure efficient NMD; and UFP2 functions to support UPF1's RNA helicase and mRNP remodeling activities.

## MATERIALS AND METHODS

### Protein production

pProExHTb plasmids (Invitrogen) encoding near full-length UPF2L (121–1227) (Neu-Yilik et al. 2017), MIF4G-D3 (761–1054) (Kadlec et al. 2004), MIF4G-D1 (120–486), MIF4G-D2 (455–757), MIF4G-D1–2 (120–757), and MIF4G-D2–3 (455–1054) constructs (Clerici et al. 2014) all comprise a C-terminal TEV-cleavage site and a His_6_ tag. The plasmids were transformed into *Escherichia coli* BL21 Rosetta (DE3) (Novagen) cells and grown in LB media supplemented with 100 μg/mL ampicillin to an OD_600 nm_ of 0.6–0.8, induced with 1 mM isopropyl 1-thio-β-d galactopyranoside, and incubated overnight (∼16 h) at 20°C. After harvesting by centrifugation at 5000*g* for 15 min at 4°C, cell pellets were resuspended in 25 mM HEPES (pH 7.45), 300 mM NaCl, 10 mM imidazole, 0.05% TWEEN 20, 5% glycerol supplemented with cOmplete EDTA-free Protease Inhibitor Cocktail Tablet (Roche) and Benzonase Nuclease (Merck). Cells were lysed by sonication and the cell lysate clarified by centrifugation at 45,000*g* for 60 min at 4°C. The supernatant was incubated with 5 mL of Ni-NTA resin for 1 h at 4°C. Beads were washed with 25 mM HEPES (pH 7.45), 300 mM NaCl, 30 mM imidazole, and 5% glycerol buffer. The proteins were eluted by incubation with 25 mM HEPES (pH 7.45), 300 mM NaCl, and 5% glycerol buffer containing increasing concentrations of imidazole from 50 to 250 mM. Eluted fractions containing the protein of interest were pooled and buffer-exchanged to 25 mM HEPES (pH 7.45), 150 mM NaCl, 1 mM TCEP, and 5% glycerol. The sample was applied onto a HiTrap Heparin column (Cytiva) using a 50 mL superloop (GE HealthCare). After washing, the proteins were eluted via a linear gradient of 150–1000 mM NaCl over 20 column volumes. Elution fractions were analyzed by SDS-PAGE. Fractions containing the protein of interest were pooled and concentrated to ∼8–10 mg/mL using an appropriate molecular weight cutoff concentrator (Merck). Concentrated protein fractions were further purified by size exclusion chromatography (SEC) using a Superdex 75 Increase 10/300 GL column or a Superdex S200 10/300 GL column (GE HealthCare), depending on the molecular weight of the UPF2 variant. Columns were equilibrated with SEC buffer (25 mM HEPES at pH 7.45, 300 nM NaCl, 1 mM TCEP, and 5% glycerol). Peak fractions were analyzed by SDS-PAGE, pooled and quantified using a NanoDrop One spectrophotometer (Thermo Scientific), followed by concentrating to ∼8–10 mg/mL. Extinction coefficient and molecular weight were determined using ExPASy: SIB bioinformatics resource portal (https://www.expasy.org). Concentrated protein was flash-frozen in aliquots at −80°C for storage.

UPF1 (residues 115–914) was expressed and purified as previously described (Deniaud et al. 2015). UPF3B comprising residues 41–262, consisting of the RNA-recognition motif, NOPS-Like domain and the first coiled coil-like domain, was expressed in *E. coli* and purified as described (Bufton et al. 2022).

### Nucleic acid oligonucleotides

The sequences of the oligonucleotide constructs used in this study are listed in Supplemental Table S1. The DNA and RNA oligonucleotides are based on a 24 nt oligonucleotide sequence previously utilized (Neu-Yilik et al. 2017). HEX-labeled probes were ordered from Eurofins Genomics UK and dissolved using 100 μL of Milli-Q water to generate a high stock concentration. Oligomer concentrations were determined using a NanoDrop One spectrophotometer (Thermo Scientific). Double-stranded 24 nt DNA and RNA (ds-DNA/ds-RNA) were prepared by mixing the complementary oligonucleotides in a 1:1 molar ratio, heating to 65°C, and slow cooling overnight to room temperature in a polystyrene container. Resulting stock concentrations of oligomers were aliquoted into small volumes and flash-frozen at −80°C for storage.

### Electrophoretic mobility shift assay

EMSA experiments were performed using HEX-labeled RNA oligonucleotides (Supplemental Table S1) diluted to 250 nM in 25 mM HEPES (pH 7.45), 100 mM NaCl, and 5% glycerol. Excess concentration of probe was necessary to detect multiple band shift species (Bufton et al. 2022). Serial dilutions of UPF2L, MIF4G-D3, UPF3B (residues 41–262), and/or UPF1 were performed using buffer containing 25 mM HEPES (pH 7.45), 300 nM NaCl, 5% glycerol, and 2 mM β-mercaptoethanol or 1 mM TCEP. Proteins, RNA, and native sample buffer (Thermo Scientific) were mixed and incubated for 1 h on ice before loading onto Native Novex WedgeWell 4%–20% Tris-glycine gels in Tris-glycine native running buffer (Invitrogen). Gels were run for 45 min at 150 V and 4°C before detecting the HEX label using a Typhoon FLA 9500 instrument (GE HealthCare) at 532 nm wavelength and BPG1 emission filter. EMSA gels were stained for protein using Coomassie Brilliant Blue.

### CD spectroscopy

The 24 nt oligonucleotide ss-RNA and hp-RNA were dissolved in TE buffer (10 mM Tris–HCl at pH 7.5 and 1 mM EDTA) to achieve a sample concentration of 100 μM and used in the experiments at 4 μM final concentration. UPF2L was used at 10 μM concentration in a 200 μL volume of 20 mM HEPES (pH 7.5) and 150 mM NaF. The CD spectra were recorded over a 200–340 nm wavelength range at 20°C, with a 1 nm data interval, using a Jasco J-810 Spectropolarimeter, in 1 mm path-length cell. Scanning was performed at 50 nm/min and eight accumulations were recorded for each spectrum. The buffer spectrum was subtracted from the sample spectra. The spectra of RNA only were further subtracted from the UPF2L/RNA spectra for analysis of RNA tertiary structure changes. Vice versa, the spectrum of UPF2L was subtracted from the UPF2L/RNA spectra for analysis of protein structural changes. Analysis of spectra was performed using GraphPad Prism7 software.

### Fluorescence spectroscopy

Fluorescence spectroscopy was carried out using a CLARIOstar Plus plate reader (BMG Labtech). The molecular beacon was dissolved in TE buffer (10 mM Tris–HCl at pH 7.5 and 1 mM EDTA) to obtain a concentration of 100 μM, following the manufacturer recommendations. RNA samples at a concentration of 250 nM were incubated for 15 min at 25°C in a Corning 384 well microplate (low volume black polystyrene, flat bottom). Fluorescence excitation and emission were recorded at 539 and 559 nm, respectively. Subsequently, indicated concentrations of UPF2L were added in the wells, and the incubation was continued for 1 h at 25°C. Experiments were performed in duplicate in 20 μL final volume. The obtained data were plotted after subtracting the background fluorescence signals using GraphPad Prism7 software.

### Fluorescence anisotropy measurements

Fluorescence anisotropy analyses were performed using a Jobin Yvon Fluorolog-3 (HORIBA Scientific) instrument. HEX-labeled oligonucleotides (Supplemental Table S1) were diluted to 10 nM in 150 µL of assay buffer (25 mM HEPES at pH 7.45, 150 mM NaCl, and 5% glycerol) and placed in a 10 mm × 2 mm Quartz Suprasil cuvette (Hellma Analytics). Calibration and a zero-point reading were measured for excitation at 530 nm and emission at 550 nm. One microliter of UPF2L or MIF4G-D3 was added stepwise at different concentrations to achieve a serial dilution of protein within the cuvette. Measurements were performed using an integration time of 0.5 sec over four accumulations and repeated in triplicate for each concentration. Assays were repeated in triplicate. Resulting signals were plotted using GraphPad Prism7 with the equation:

*Y*= *B*_max_ × [*X*]/(*K*_D_ + [*X*]) + *C*

(referring to a one site specific binding [where *B*_max_ is the total number of specific binding and *X* is the concentration of free protein] used to calculate the equilibrium dissociation constant [*K*_D_] and standard deviation).

### Sample preparation and negative-stain EM

Purified UPF2L in SEC buffer (25 mM HEPES at pH 7.45, 150 mM NaCl, 1 mM TCEP, 2 mM MgCl_2_, and 5% glycerol) was used for negative-stain EM sample preparation. Complex formation was achieved by adding a fourfold molar excess of hp-RNA or ss-RNA to UPF2L and incubating for 1 h on ice. The sample was centrifuged at 16,200*g* for 10 min and immediately used for grid preparation.

Carbon-coated copper 300 mesh grids (Electron Microscopy Sciences) were glow-discharged (15 sec at 15 mA) on a Leica EM ACE600 instrument (Leica Microsystems). Five microliters of 0.01 mg/mL UPF2L, 0.01 mg/mL UPF2L/ss-RNA complex, and 0.035 mg/mL UPF2L/hp-RNA complex was applied to the grids and incubated for 1 min before blotting with Whatman filter paper (GE HealthCare). Five microliters of 3% (w/v) uranyl-acetate was applied and immediately blotted before applying an additional 5 µL of uranyl-acetate and incubating for 15–30 sec followed by blotting. Data were collected using a FEI T20 200 kV Twin lens TEM microscope (within the Wolfson Bioimaging Suite, University of Bristol) and imaged at 68,000× magnification corresponding to a pixel size of 1.44 Å using a Ceta 4kx4k charge-coupled device camera (FEI).

### Negative-stain EM image processing

Images were processed using CryoSPARC (Punjani et al. 2017) and RELION 3.1 (Scheres 2012) software packages. UPF2L micrographs were imported without contrast transfer function correction. Particles (2500) were manually picked and used for two rounds of 2D classifications using 25 classes. Eight classes were selected as templates for autopicking, where 95,478 particles were picked. The autopicked particles were subjected to multiple rounds of 2D classifications. A subset of class selections was used to generate final 2D class averages. Next, 532,115 particles were initially picked from micrographs with UP2L/hp-RNA sample. Then, 224,197 particles were picked from UPF2L/ss-RNA micrographs. Two-dimensional class averages containing most particles are shown in Figure 5 and Supplemental Figure S6.

### RNA and protein structure predictions

The RNA 2D models were predicted using the Vienna RNA web suite (Lorenz et al. 2011) and MXfold2 (Sato et al. 2021). The RNA 3D models were predicted using 3dRNA/DNA (Zhang et al. 2022) and trRosettaRNA (Wang et al. 2023). To predict 2D and 3D dimer models of ss-RNA, a U_10_ or A_10_ linker sequence was inserted linking two ss-RNA molecules. This linker was then deleted from 2D and 3D models for visualization (Sato et al. 2021).

The UPF2L structure predictions were acquired from the ColabFold: AlphaFold2 Protein Structure Database (Mirdita et al. 2022). The five output predicted models were automatically ranked (1—best to 5—worst) based on the highest average predicted local distance difference test score for single structures and the pTM score for complexes. Subsequently, input UPF2L structures were relaxed to a low energy state according to backbone, relaxation, sidechain repacking, and bond strength using the Relax application, as part of the Rosetta software suite (https://www.rosettacommons.org) (Tyka et al. 2011). Models were subjected to three cycles of relaxing to generate five models, ranked by total score, relating to the energy values from 19 tested terms including the attraction and repulsion energy between atoms, intermolecular bond forces (Van der Waals, hydrogen bond, and disulfide bond), and side chain torsion angles (for full list of test terms, see Alford et al. 2017).

## DATA DEPOSITION

All data for this manuscript are contained within the main article and Supplemental Material.

## SUPPLEMENTAL MATERIAL

Supplemental material is available for this article.

## Figures and Tables

**Jenn-Yeu Alvin Szeto:**
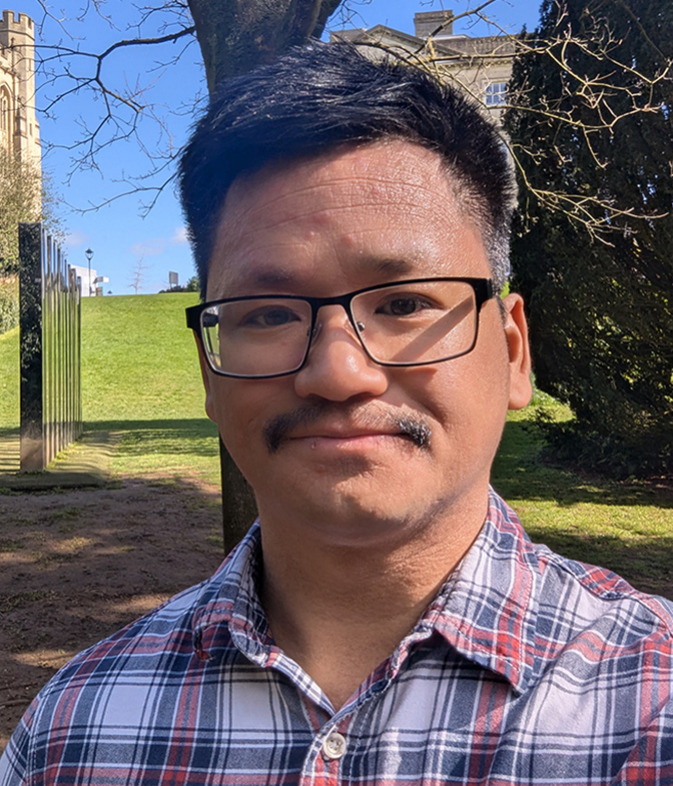


**Mirella Vivoli Vega:**
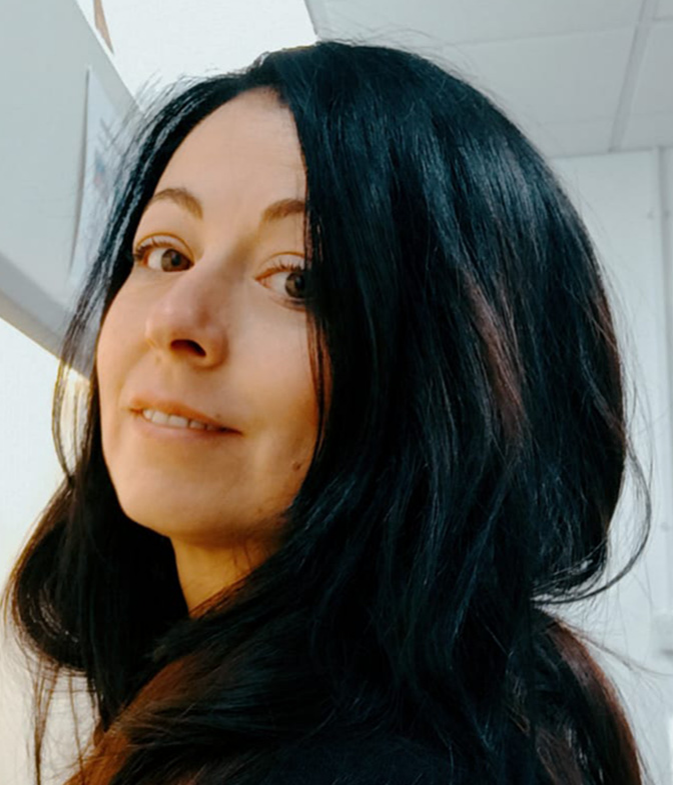


## MEET THE FIRST AUTHORS

***Meet the First Author(s)* is an editorial feature within *RNA*, in which the first author(s) of research-based papers in each issue have the opportunity to introduce themselves and their work to readers of *RNA* and the RNA research community. Jenn-Yeu Alvin Szeto and Mirella Vivoli Vega are co-first authors of this paper, “Dynamic RNA binding and unfolding by nonsense-mediated mRNA decay factor UPF2.” Jenn-Yeu is a postdoctoral researcher with an interest in protein structure, engineering, and drug design. Jenn-Yeu completed his PhD in 2023 with Prof. Christiane Schaffitzel as part of his BBSRC-funded SWBio DTP scholarship at the University of Bristol. Jenn-Yeu's thesis focused on the interactions between UPF proteins and nucleic acids using structural and biophysical approaches. Mirella is a Senior Research Fellow and former Marie Skłodowska-Curie Fellow at the University of Bristol. Mirella's research has focused on dissecting the role of UPFs in the NMD pathway—a crucial surveillance mechanism conserved across all eukaryotes, including humans.**

**What are the major results described in your paper and how do they impact this branch of the field?**

This study reveals that UPF2, a core component of the NMD pathway, directly binds and remodels RNA, preferentially interacting with ss-RNA through its MIF4G domains—particularly domain 3. UPF2 not only unfolds structured RNAs but also undergoes a conformational change upon RNA binding. These activities persist in the presence of UPF1 and UPF3B, suggesting a cooperative role in stabilizing UPF1 on target mRNAs and enhancing mRNP remodeling. Our findings uncover a previously unrecognized RNA-dependent function of UPF2 that could play a role in nonsense mRNA recognition and degradation.

**What led you to study RNA or this aspect of RNA science?**

**MVV:** As a protein scientist, I've always been fascinated by the intricacies of the translation mechanism. That curiosity naturally led me upstream, to RNA and the remarkable layers of control that determine protein expression. I was particularly drawn to the elegant pathways that ensure mRNA quality, especially NMD.

**JYAS:** I had a keen interest in protein structure, function, and interaction relationships since my undergraduate studies at Cardiff University. When looking for a PhD project, I wanted one that would fit these criteria. Despite being discovered over 40 years ago, many questions remain regarding molecular mechanisms and interactions between NMD factors and RNA. Using structural, biochemical, and biophysical techniques, I hoped I could contribute to answering some of these questions.

**During the course of these experiments, were there any surprising results or particular difficulties that altered your thinking and subsequent focus?**

**JYAS:** A surprising moment during my PhD came when processing the relaxed models of the UPF2 structure generated by AlphaFold2. They bore a striking resemblance to the negative-stain micrographs and 2D class averages. This suggested that UPF2 is flexible, which contrasted with previously published structural data, showing that UPF2 displays rigid assemblies and compact ring-like structures in the absence of RNA.

**What are some of the landmark moments that provoked your interest in science or your development as a scientist?**

**MVV:** I grew up in a peaceful small town in southern Italy, surrounded by the beauty of nature. My father instilled in me three core values that continue to guide my life: Questioning, Exploring, and Learning. Raised in an international family, I was taught a strong work ethic and a deep appreciation for nature. From a young age, I was captivated by what I like to call “thinkering”—a portmanteau of thinking and tinkering. I often placed natural objects under the microscope, curious to understand how they worked, and carefully recorded my observations. My interest in protein science began during my bachelor's and master's studies in molecular biology at the University of Rome Sapienza. During my PhD in Biochemical Sciences, I became utterly—almost addictively—fascinated by the intricate world inside the cell. That journey led me to specialize in protein science with the goal of using my knowledge to advance drug discovery and help combat disease.

**JYAS:** I grew up watching *Walking with Dinosaurs, Blue Planet*, and *MythBusters*. I loved seeing what was out there, what is currently out there, and entertaining ways to test myths and legends through scientific experiments. *MythBusters*, along with science practicals, developed my critical thinking in a fun and engaging way. When it came to undergraduate studies, I vividly remember the first time I saw the cartoon and surface structures of proteins, learning about catalytic triads of trypsin and chymotrypsin and attending a workshop on how to use PyMOL. This developed my interest in structure and function relationships, particularly in structure determination techniques such as X-ray crystallography and cryo-EM.

**If you were able to give one piece of advice to your younger self, what would that be?**

**JYAS:** I'd probably say not to compare yourself to others. This applies in general life as well as in science. For example, everyone is on their own career path, so if someone gets a PhD or job before you, doesn't mean you're falling behind. The same thing applies to laboratory work too—just because someone gets good results doesn't mean you're doing anything wrong.

**Are there specific individuals or groups who have influenced your philosophy or approach to science?**

**MVV:** First and foremost, my father instilled in me the belief that education is the only true path to freedom. He encouraged me to become not just a successful person, but a great one—great in curiosity, integrity, and knowledge. His words continue to echo in everything I do: learn as much as you can, because that is how you become truly free. Scientifically, I've been profoundly influenced by Richard Feynman and Marie Skłodowska-Curie. Feynman taught me that science is not about memorizing facts, but about joyful discovery, asking questions fearlessly, and finding beauty in understanding how the world works. His playful yet rigorous mindset reminds me that passion and precision can go hand-in-hand. Marie Curie, on the other hand, embodies perseverance, humility, and impact. She faced immense barriers yet never allowed them to limit her scientific vision. Her dedication to using science to improve lives—often at great personal cost—continues to inspire me to pursue research that is not only intellectually meaningful but also socially relevant. Both of them remind me that science isn't just a career—it's a way of living with wonder and integrity.

**How did you decide to work together as co-first authors?**

**MVV:** Alvin did tremendous work studying the role of the UPF2 protein and its ability to unwind RNA molecules throughout his PhD. Given my specialization in biophysics, we realized that combining our strengths would enhance the depth and robustness of our findings. We decided to work together as co-first authors to integrate our complementary expertise and reinforce the scientific impact of the study.

**JYAS:** Mirella has always been helpful and supportive, with experience in a wide range of skills. Her assistance in following biophysical experiments was invaluable to validating the unwinding behavior of UPF2 toward structured RNA.
